# Darobactin B Stabilises a Lateral‐Closed Conformation of the BAM Complex in *E. coli* Cells

**DOI:** 10.1002/anie.202218783

**Published:** 2023-05-31

**Authors:** Samuel F. Haysom, Jonathan Machin, James M. Whitehouse, Jim E. Horne, Katherine Fenn, Yue Ma, Hassane El Mkami, Nils Böhringer, Till F. Schäberle, Neil A. Ranson, Sheena E. Radford, Christos Pliotas

**Affiliations:** ^1^ Astbury Centre for Structural Molecular Biology School of Molecular and Cellular Biology University of Leeds Leeds LS2 9JT UK; ^2^ Astbury Centre for Structural Molecular Biology School of Biomedical Sciences University of Leeds Leeds LS2 9JT UK; ^3^ School of Biological Sciences, Faculty of Biology, Medicine and Health Manchester Academic and Health Science Centre The University of Manchester Manchester M13 9PT UK; ^4^ Manchester Institute of Biotechnology The University of Manchester Manchester M1 7DN UK; ^5^ School of Physics and Astronomy University of St. Andrews St. Andrews KY16 9SS UK; ^6^ Institute for Insect Biotechnology Natural Product Research Justus-Liebig-University Giessen Ohlebergsweg 12 35392 Giessen Germany; ^7^ German Center for Infection Research (DZIF) Partner Site Giessen-Marburg-Langen Ohlebergsweg 12 35392 Giessen Germany; ^8^ Natural Product Department Fraunhofer-Institute for Molecular Biology and Applied Ecology (IME) Ohlebergsweg 12 35392 Giessen Germany

**Keywords:** BAM, Cryoem, Darobactin, In-Cell EPR, PELDOR

## Abstract

The β‐barrel assembly machinery (BAM complex) is essential for outer membrane protein (OMP) folding in Gram‐negative bacteria, and represents a promising antimicrobial target. Several conformational states of BAM have been reported, but all have been obtained under conditions which lack the unique features and complexity of the outer membrane (OM). Here, we use Pulsed Electron‐Electron Double Resonance (PELDOR, or DEER) spectroscopy distance measurements to interrogate the conformational ensemble of the BAM complex in *E. coli* cells. We show that BAM adopts a broad ensemble of conformations in the OM, while in the presence of the antibiotic darobactin B (DAR‐B), BAM′s conformational equilibrium shifts to a restricted ensemble consistent with the lateral closed state. Our in‐cell PELDOR findings are supported by new cryoEM structures of BAM in the presence and absence of DAR‐B. This work demonstrates the utility of PELDOR to map conformational changes in BAM within its native cellular environment.

## Graphical abstract

The darobactins are promising new antibiotics that inhibit the BAM complex, an essential membrane‐protein foldase in Gram‐negative bacteria. Here, we use EPR spectroscopy (PELDOR, or DEER) distance measurements to monitor the effect of darobactin B on BAM in *E. coli* cells, showing it stabilises BAM in a lateral gate closed conformational state. Our results provide the first direct evidence for conformational selection by a darobactin in the cellular context.

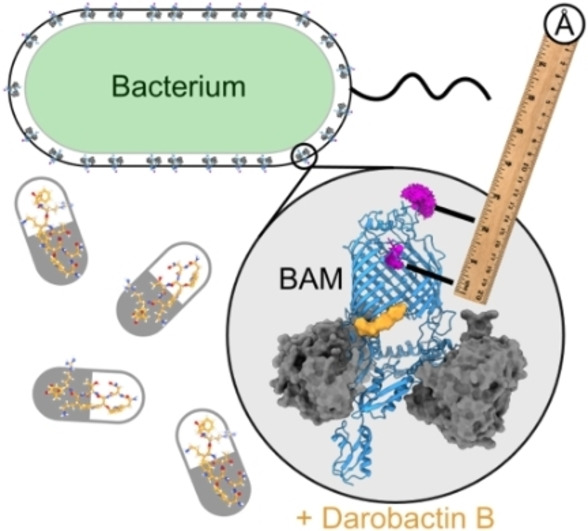

## Introduction

**Figure 1:**
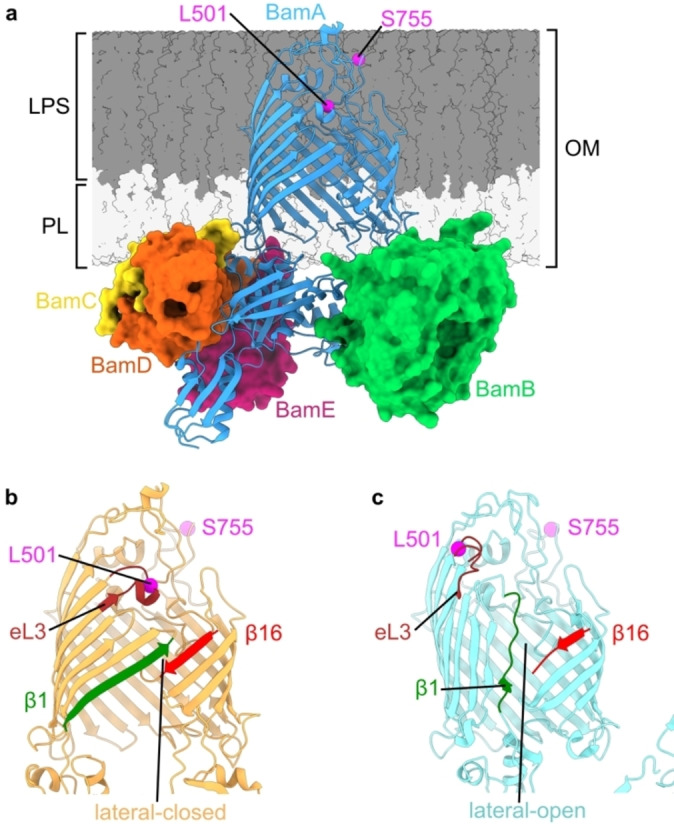
Conformational heterogeneity of the BAM complex. a) Structure of the full BamABCDE complex (PDB 5D0O).^[5]^ Positions of L501 and S755, labelled in the L501R1‐S755R1 spin pair discussed in this manuscript, are indicated (magenta). Relative height of LPS and phospholipid (PL) portions of the OM are indicated. BAM adopts b) lateral‐closed (PDB 5D0O)^[5]^ and c) lateral‐open (PDB 5LJO)^[6]^ conformations, distinguished by closing or opening of a lateral‐gate between β‐strands β1 and β16 of the BamA β‐barrel. Opening also results in movement of the N‐terminal barrel face involving β‐strands β1‐β6, and the extracellular loops (eLs) between them. Extracellular loop 3 (eL3, brown) is highlighted as movement of this loop (which contains L501) leads to distance changes for the L501R1‐S755R1 pair. The BamBCDE subunits are hidden in (b) and (c) for clarity.

The folding of outer membrane proteins (OMPs) in Gram‐negative bacteria is facilitated by the β‐barrel assembly machinery (BAM) (Figure 1a). This is an essential process for the bacterial cell, and BAM therefore represents a promising target for new antimicrobials.[^1^, ^2^, ^3^] BAM is thought to exist in two major conformations (Figure 1b and c), defined by closing (lateral‐closed, Figure 1b) or opening (lateral‐open, Figure 1c) of a membrane‐facing gate in the core component BamA, itself an OMP. Although multiple structures of both states have been solved by X‐ray crystallography and cryogenic Electron Microscopy (cryoEM), these have been obtained using membrane mimetics such as detergents, SMALPs, or nanodiscs.[^4^, ^5^, ^6^, ^7^] They are therefore determined in the absence of important features of BAM's environment in the OM including a) the lipopolysaccharide (LPS) layer and consequent unique OM asymmetry (Figure 1a), b) the lattice‐like packing of OMPs in the OM,^[8]^ and c) substrates, chaperones, and other interaction partners which define BAM's functional roles.^[9]^ To date the impact of these factors on BAM, and its native conformational ensemble in the OM, is unknown. Transitioning between lateral open and closed states is proposed to be essential for BAM function,[^7^, ^10^] with multiple antimicrobials shown to bias the conformational equilibrium in vitro.[^2^, ^3^, ^7^] These include darobactin A (DAR‐A), a member of a promising class of broad spectrum antibiotics against major Gram‐negative pathogens[^11^, ^12^, ^13^] and the related, dynobactin.^[14]^ In existing structures, DAR‐A binds to the first β‐strand (β1) of BamA, blocking substrate binding, and locking the complex in a lateral‐closed conformation.[^2^, ^12^, ^13^] However, it is currently unknown if this conformational change also occurs within cells, and hence the mechanism of action of BAM‐targeted antibiotics in vivo remains unresolved.

**Figure 2:**
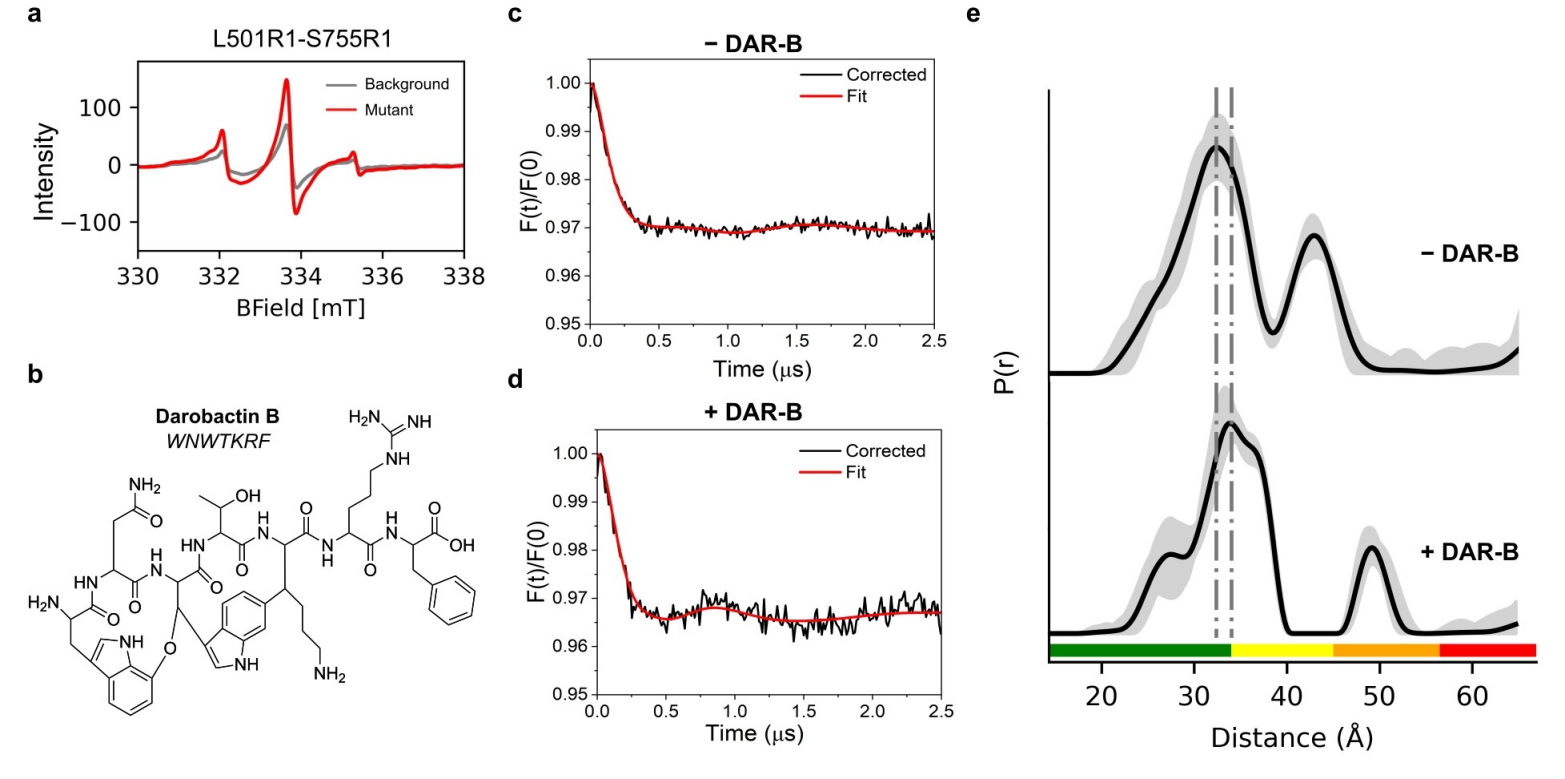
Darobactin B (DAR‐B) alters the BAM conformational ensemble in whole cells. a) Xband room temperature cwEPR spectra of MTSSL labelled cells containing the L501R1‐S755R1 spin pair (red). Background signal from labelled cells expressing Cys‐free BAM is also shown (grey). b) Chemical structure and peptide formula of Darobactin‐B (DAR‐B). c) Experimental background‐corrected (black line) and fitted (red line) PELDOR traces for MTSSL labelled BAM L501R1‐S755R1, measured in cells. d) Experimental background‐corrected PELDOR traces for MTSSL labelled BAM L501R1‐S755R1, measured in cells after incubation with DAR‐B. For (c) and (d) PELDOR traces from MTSSL labelled cells expressing Cys‐free BAM under the same conditions (−/+DAR‐B respectively) were used for experimental background‐correction. These are shown in Figure S5a. Additional background correction analysis is presented in Figures S6 and S7 and discussed in Methods. PELDOR traces of single Cys BAM variants (L501R1 and S755R1), using 1 to 1 weighting in DeerAnalysis and corresponding experimental background corrected data (+DAR‐B) are also shown in Figure S6c–f, yielding similar distance distributions to Cys‐free BAM background. e) PELDOR‐derived distance distributions for BAM L501R1‐S755R1 +/−DAR‐B, showing the shifting of the distribution upon addition of the antibiotic. For PELDOR, the mean distance (black) and 2σ confidence intervals (shaded grey areas) show the mean ±2*σ* confidence intervals of the measured distributions calculated using the validation tool in DeerAnalysis (Methods). Traffic light indicates reliability of PELDOR distribution: (green, shape reliable; yellow, mean and width reliable; orange, mean reliable; red, no quantification possible). Dashed lines indicate positions of peaks in PELDOR distance distributions. Y‐axis for distance distributions indicate the probability density *P*(*r*).

PELDOR, also known as Double Electron‐Electron Resonance (DEER) spectroscopy combined with site‐directed spin labelling (SDSL) is a powerful tool in the investigation of protein structure and dynamics.[^15^, ^16^, ^17^, ^18^] The technique can measure distances between paramagnetic centres that lie 20–80 Å apart, and in special cases inter‐spin distances extending to 160 Å[^19^, ^20^] Over the past decades, most distance measurements have been made using nitroxide‐based organic radicals,^[21]^ but the increasing interest in investigating proteins in their native environment, including in cells, has extended spin label libraries to include new labels based on paramagnetic metal ion complexes.[^21^, ^22^, ^23^, ^24^, ^25^, ^26^, ^27^, ^28^, ^29^, ^30^] For integral membrane proteins, PELDOR has mostly relied on distance measurements between spin‐labels that are covalently attached to engineered cysteines on protein sites. The method has been used to decipher membrane transport and channel gating dynamics by providing quantitative information on conformational changes, either in combination with structural information provided by cryoEM and/or X‐ray crystallography, or as a stand‐alone method.[^31^, ^32^, ^33^, ^34^, ^35^, ^36^, ^37^, ^38^, ^39^, ^40^, ^41^, ^42^] In the latter case, PELDOR can take advantage of static Alphafold models to interpret the conformational changes that occur.[^33^, ^36^] Owing to its sensitivity, speed and flexibility compared to other structural methods, PELDOR has been used to describe conformational transitions in OMPs in vitro,^[43]^ and also in situ in purified OM extracts or in *E. coli* cells.[^44^, ^45^] These measurements underlined the importance of the native environment in determining the conformation(s) of a membrane protein. For example, the OMPs, BtuB (in‐cell and purified OM extracts) and BamA, the core OMP subunit of the BAM complex, (in purified OM extracts) have different conformational ensembles in biological membrane preparations versus measurements in detergent.[^46^, ^47^, ^48^, ^49^, ^50^] Here, we implemented PELDOR distance measurements on the intact, five component, BAM complex (BamABCDE) in *E. coli* cells by engineering Cys‐pairs at different positions in its core BamA subunit (Figure 1 and S1). We show that the antibiotic darobactin B (DAR‐B) (Figure 2b), a homologue of DAR‐A,^[13]^ also binds in the lateral‐closed state in vitro and induces closure of the BAM lateral gate in vivo, showcasing the biological relevance of its mechanism of action in a cellular context. By contrast, in the absence of DAR‐B, BAM exhibits a broad distribution of distances, which point towards a highly dynamic and heterogeneous conformational ensemble. In support of our in‐cell PELDOR findings we solve new cryoEM structures of BAM in the presence and absence of DAR‐B to enable direct comparison and accurate modelling of the derived in‐cell PELDOR distance distributions.^[51]^

## Results and Discussion

We first identified sites that are accessible to spin labels and would enable the lateral‐open/lateral‐closed BAM structures to be resolved using PELDOR. As our chosen label (methanethiosulfonate) (MTSSL) is reduced upon entering the bacterial periplasm,^[45]^ sites were restricted to the extracellular loops (eL) of BamA. In X‐ray and cryoEM structures, opening of BAM's lateral‐gate leads to an outward swinging motion of the six N‐terminal β‐strands of the barrel relative to the ten C‐terminal strands and their connecting loops. Pairs were thus chosen with one position on an N‐terminal loop and the other on a C‐terminal loop (Figure S1). Modelled distance changes ranged from ca. 1–2 nm between the selected sites, dependent on whether the lateral gate is open or closed. Using these criteria, we designed four pairs: T434R1‐Q801R1, E435R1‐S755R1, E435R1‐Q801R1 and L501R1‐S755R1 (Figure S1). R1 refers to the modification with MTSSL on Cys residues engineered into the protein at the sites of interest.

To test whether each of the selected sites could be labelled with MTSSL, single Cys residues were introduced into a BAM complex in which the two natural Cys of BamA were replaced with Ser (BamA C690S C700S, named Cys‐free BAM hereafter) (note that BamBCDE contain no free Cys). Cys‐free BAM can complement wild‐type BamA in vivo and shows similar OMP folding activity to BAM in vitro.[^6^, ^7^, ^10^] Cells were then labelled with MTSSL and the extent of labelling for the different sites measured using continuous wave EPR (cwEPR). In each case, the EPR signal for cells expressing each single Cys variant was compared to those of cells expressing Cys‐free BAM to assess background signal resulting from non‐specific labelling of OM components. Most selected sites exhibited significant additional labelling over background (Figure S2), indicating that they are accessible to the spin label. However, variable extents of labelling were observed for different sites, presumably reflecting differential accessibility of the engineered thiols in the complex OM environment. Indeed, OMPs in the OM form a rigid, tightly‐packed lattice, mediated by protein‐lipid interactions.^[8]^ This, combined with the height of the LPS chains, which increases OM thickness (Figure 1a), could plausibly restrict access of the label to the extracellular regions of BamA in the OM.

Following verification of spin labelling of both single‐ and pair‐labelled Cys variants, Q‐band PELDOR distance measurements were performed for each Cys pair, which enabled calculation of spin labelling efficiencies based on the respective modulation depths. This analysis yielded labelling of ca. 20–45 % (Table S1), similar, albeit slightly lower, than results obtained previously using purified BamA in extracted OMs.^[46]^ Cys‐free BAM was also measured under identical conditions to obtain a PELDOR trace for the respective experimental background (Methods). This was in order to a) test for any non‐specific distances present and b) allow for determination of experimental background‐corrected PELDOR traces from which reliable distance distributions could be derived. western blot analysis showed that each Cys‐pair construct was expressed in the bacteria (Figure S3). However, three of the spin labelled pairs (T434R1‐Q801R1, E435R1‐S755R1, and E435R1‐Q801R1) presented no clear dipolar modulation in the raw data (Figure S4a), and despite deviating significantly from background, resulted in broad distributions (Figure S4b). Such broad distributions are consistent with BAM occupying a heterogeneous ensemble of conformations in the native OM. However, it was not possible to draw any additional reliable conclusions from these spin pairs and the associated data were excluded from further analysis.

The spin pair variant BAM L501R1‐S755R1 showed significant additional labelling compared to the Cys‐free BAM background (Figure 2a) and signs of shallow dipolar modulation in the raw PELDOR data which is more enhanced in both the experimentally and model‐free background‐corrected data (Figure 2c, and Figures S5 and S6). We therefore decided to take this pair forward to test the effect of the DAR‐B (Figure 2b) antimicrobial on BAM in *E. coli* cells. Labelled cell suspensions containing the L501R1‐S755R1 pair were incubated with either DAR‐B, dissolved in DMSO (+DAR‐B) or DMSO alone (−DAR‐B), before being snap freezing for PELDOR. DAR‐B has been shown to bind to the lateral‐closed state of the BamA β‐barrel in vitro.^[13]^ In the absence of DAR‐B we obtained a broad, but defined, distance distribution, which did not alter in width and mean distance values irrespective of the data analysis or computational method used, and despite an effect on modulation depth depending on the background correction, leading to a clear consensus (Figure 2c, e, and Figures S5c, S6 and S7b). This distribution is consistent with BAM adopting a broad conformational ensemble in cells, in accord with the other three pairs we considered above. Consistent results were obtained on different days and with different batches of cells (Figure S8). We next tested the effect of adding the antibiotic DAR‐B on BAM‐MTSSL labelled cell suspensions containing the L501R1‐S755R1 pair, using Cys‐free BAM or single Cys variants as experimental background. Addition of DAR‐B to L501R1‐S755R1 led to a change in the PELDOR raw and experimental background corrected data, whether we used a Cys‐free BAM or single Cys mutant background (i.e. L501R1 and S755R1 mixture weighted 1 to 1 using DeerAnalysis), which both exhibited clear dipolar modulations (Figure 2d, and Figures S5b and S6), resulting in a substantial shift relative to the distance distribution obtained in the absence of DAR‐B (Figure 2e, and Figures S5c and S7a). Conversely, no change in the raw data was seen for Cys‐free BAM (Figure S5a) indicating that the observed effect is specific to BAM. DAR‐B therefore appears to restrict BAM′s conformational ensemble in cells.

**Figure 3:**
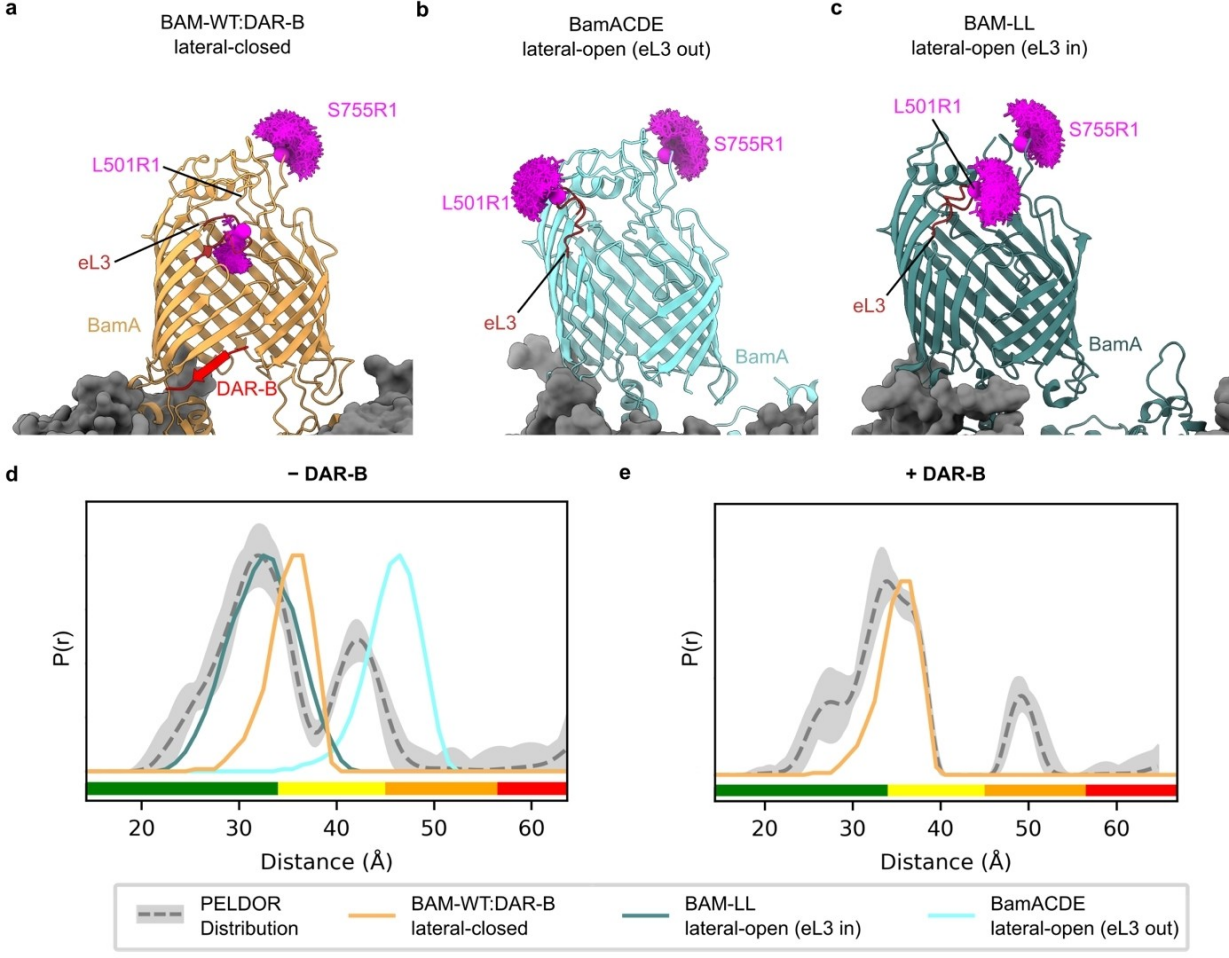
Determination of the conformation of BAM in the presence and absence of DAR‐B. a) Lateral‐closed conformation of BAM in complex with DAR‐B determined here using cryoEM (PDB: 8BVQ), with eL3 pointing into the barrel (orange). b) Lateral‐open conformation of BamACDE determined using X‐ray crystallography (PDB: 5D0Q)^[5]^ with eL3 pointing out of the barrel (light blue). This structure is used for modelling the distance between L501R1 (which lies in eL3) and S755R1, as it has clear density for L501, while this residue, along with others in eL3 are absent in other lateral‐open BAM structures, including the cryoEM structure of apo‐BAM determined in this study (Figure S14). c) Lateral‐open like conformation of a lid‐locked BAM (PDB: 7NBX)^[7]^ with eL3 pointing into the barrel (dark green). Predicted MTSSL ensembles for L501R1‐S755R1 are indicated for each structure (magenta). d) Comparison of observed PELDOR distribution for L501R1‐S755R1 −DAR‐B (grey) to the predicted distance distribution for all structures in (a)–(c). The distribution is consistent with a mixture of lateral‐open (eL3 in) and lateral‐closed conformations. e) Comparison of observed PELDOR distance distribution for L501R1‐S755R1 +DAR‐B (grey) to the predicted distance distribution in silico for the lateral‐closed BAM‐WT : DAR‐B structure, showing good agreement of this prediction with the observed distribution. For PELDOR, the mean distance (dashed grey) and 2*σ* confidence interval (light grey) (defines as in Figure 2) are shown. Traffic light indicates reliability of PELDOR distribution: (green, shape reliable; yellow, mean and width reliable; orange, mean reliable; red, no quantification possible). Y‐axis for distance distributions indicate the probability density *P*(*r*).

To better understand the structural transitions that BAM undertakes in the absence and presence of DAR‐B we performed in silico modelling on existing BAM structures. As no structure of DAR‐B bound to the complete BAM complex has been reported, we also solved cryoEM structures of wild‐type BAM (BAM‐WT) in n‐dodecyl‐β‐D‐maltoside (DDM) micelles in the presence or absence of DAR‐B, reaching resolutions of 3.3 Å (+DAR‐B) and 3.5 Å (−DAR‐B) (Figures S9–S12). In the absence of DAR‐B, our new *apo*‐BAM‐WT structure (Figure S9), which is currently the highest resolution structure of this complex, is in the lateral‐open conformation, consistent with previous cryoEM structures in detergent^[6]^ and nanodiscs^[4]^ (Figure S11). However, in the BAM‐WT : DAR‐B complex, binding of DAR‐B to β1 of BamA stabilises the lateral‐closed state (Figure 3a, and Figures S10 and S11). DAR‐B therefore promotes a transition to the lateral‐closed conformation in vitro, as observed for equivalent BAM structures (in detergent micelles) bound to the darobactin variants DAR‐A and DAR‐9[^2^, ^12^, ^13^] (Figure S11). DAR‐B formed a similar binding interface to β1 of BAM to that previously observed for DAR‐B bound to BamA alone (X‐ray crystal structure) or DAR‐A bound to the intact BAM complex (cryoEM structure) (Figure S12).

Predicted distance distributions for the L501R1‐S755R1 pair in the BAM‐WT : DAR‐B cryoEM structure were generated using MtsslWizard. Strikingly, these distributions showed an excellent agreement with the experimentally obtained PELDOR distribution of this complex, consistent with DAR‐B binding causing the BamA gate to lock shut in a lateral‐closed state in the intact BAM complex in the OM (Figure 3a, e and Figure S13). Comparison with other lateral‐closed BAM structures gave similar results (Figure S13). Collectively, our PELDOR and cryoEM data are consistent with DAR‐B inducing a lateral‐closed state of BAM in vivo, indicating that its mode of action in the native cellular environment mirrors that observed in vitro.

Finally, we performed in silico modelling to explore which of the currently solved structures of BAM could rationalise the distances observed for the complex in the OM in the absence of DAR‐B (Figure 3d). By contrast with the lateral closed state of BAM/BamA in which eL3, which contains L501, is highly resolved and tucked towards the barrel in all structures (named here “eL3‐in”) (Figure 3a), eL3 is dynamically disordered in BAM lateral open states, adopting different conformations in different structures (“eL‐in” and “eL‐out”) (e.g. Figure 3b and c). In some structures, including the structure of BAM solved here, eL3 is not visible in the electron density (Figures S14 and S15). This is consistent with a conformational malleability of eL3, which has been shown previously using molecular dynamics simulations, where a disulphide lock designed to trap the eL3‐out conformation also enabled eL3‐in conformations.^[4]^ As shown in Figure 3d, the PELDOR distance distribution obtained for the *apo*‐BAM in the OM is more consistent with a lateral‐open‐eL3‐in conformation (although given some persistent longer distances involved, we cannot discount the presence of a population of eL3‐out conformations) (Figure 3b and d). For assessing reliability of these larger distances, longer time traces should be obtained to conclude the extent of presence of the BAM lateral open (eL3‐out) state in cells. However, this requires longer relaxation times, which we could not access in our experimental conditions (Figure S5). Instead, the shorter PELDOR distance distribution components observed, match well with lateral open‐eL3‐in conformations, such as that seen in BAM‐LL (a conformationally constrained BAM which contains a disulphide bond between BamA E435C S665C) (Figure 3c),^[7]^ and BAM stalled in the act of folding BamA.^[52]^ The absence (or lower extent) of the lateral‐open (eL3‐out) state (Figure S5) in the *apo*‐BAM equilibrium in cells might plausibly result from conformational restrictions imposed by the tight packing of LPS and OMPs in the OM,^[8]^ which could sterically hinder the outward movement of eL3. While the L501R1‐S755R1 distance distribution of *apo*‐BAM suggests populations of both the lateral‐closed and lateral‐open (“eL3‐in”*)* species in the OM, it is also broader than the in silico predicted distribution for these states. Hence, the conformational heterogeneity of BAM in cells may not be entirely represented by existing atomic models (although more distance measurements with other spin pairs will be needed to confirm this). Nonetheless, this proposed heterogeneity accords with current models of BAM function, wherein both conformations are adopted during the catalytic cycle.^[9]^ Given that live cells were used for our measurements, at least some BAM complexes would be expected to be actively engaged in OMP folding during our experiments.

A recent PELDOR study of the isolated BamA component of the BAM complex in purified OM extracts, showed that both the open and closed states of the lateral gate could exist, but in these preparations the closed state is predominant.^[46]^ In *E. coli* cells, BAM seems to form a more diverse ensemble of distinct conformations spanning the open‐to‐closed states that is difficult to distinguish (at least using the spin pairs chosen here). The difference in conformational diversity of BamA in purified OM extracts and BAM in *E. coli* cells presumably reflects the absence of the lipoproteins (BamBCDE) in the study of isolated BamA, without which the BAM complex is not functional.^[53]^ Indeed, a lateral‐open structure of BamA alone has never been observed in vitro, suggesting the lipoproteins enable access to this state. The lateral‐open conformation is predicted to be required for the BAM functional cycle,^[9]^ consistent with it being a state naturally adopted in the OM. Finally, and importantly, in marked contrast to conformationally diverse *apo*‐BAM, the EPR data we present here provide clear evidence that DAR‐B causes the lateral gate to shut.

## Conclusion

In summary, we report the application of PELDOR to BAM in vivo in the OM of whole bacteria. We show that a broad distribution of BAM species are observed in the OM, suggesting BAM exists in multiple conformational states, as would be expected for cells in which BAM is caught in the act of folding many different OMPs into the OM. By contrast, addition of the bactericidal agent, DAR‐B causes a switch to a more homogeneous ensemble, consistent with the lateral‐closed conformation, providing direct proof of its mode of antimicrobial action in cells. PELDOR therefore represents a powerful tool to investigate the in vivo effects of other BAM‐targeting antibiotics, several of which have also been suggested to perturb BAM's conformational equilibrium.[^3^, ^7^, ^54^, ^55^] Additionally, the technique could be used to examine the effect of altering BAM's cellular context, such as alteration of OM components, alterations in LPS, changes in OMP synthesis rates, and/or deletion of lipoprotein subunits from BAM, on the functional cycle of this essential OM protein complex.

## Experimental Section

Detailed experimental procedures are given in the Supporting Information provided with this paper.

## Conflict of interest

The authors declare no conflict of interest.

1

## Supporting information

As a service to our authors and readers, this journal provides supporting information supplied by the authors. Such materials are peer reviewed and may be re‐organized for online delivery, but are not copy‐edited or typeset. Technical support issues arising from supporting information (other than missing files) should be addressed to the authors.

Supporting Information

## Data Availability

The data that support the findings of this study are available from the corresponding author upon reasonable request.
